# Role of sodium pyruvate in maintaining the survival and cytotoxicity of *Staphylococcus aureus* under high glucose conditions

**DOI:** 10.3389/fmicb.2023.1209358

**Published:** 2023-06-19

**Authors:** Ti Chen, Huan Xu, Xiaoyan Yao, Zhen Luo

**Affiliations:** ^1^Department of Laboratory Medicine, The Third Xiangya Hospital, Central South University, Changsha, China; ^2^Department of Medical Laboratory Science, School of Medicine, Hunan Normal University, Changsha, China

**Keywords:** pyruvate, *Staphylococcus aureus*, high glucose, virulence, survival, sarA

## Abstract

Glucose is a crucial carbon source for the growth of *Staphylococcus aureus*, but an excess of glucose is detrimental and even leads to cell death. Pyruvate, the central metabolite of glycolysis, has been shown to have anti-inflammatory and antioxidant properties. This study aimed to investigate the protective effect of pyruvate on *S. aureus* under high glucose conditions. Sodium pyruvate greatly increased the cytotoxicity of *S. aureus* strain BAA-1717 to human erythrocytes and neutrophils *in vitro*. However, the cytotoxicity and survival of *S. aureus* were significantly reduced by high glucose, which was restored to normal levels by the addition of sodium pyruvate. The expression of hlg and lukS in *S. aureus* was higher in the LB-GP cultures than that in LB-G cultures, but there was no significant difference in cytotoxicity between LB-GP and LB-G cultures. Furthermore, the hemolytic activity of *S. aureus* supernatants could be inhibited by the cell-free culture medium (CFCM) of LB-G cultures, suggesting that high levels of extracellular proteases were presence in the CFCM of LB-G cultures, resulting in degradation of the hemolytic factors. The expression of sarA, which negatively regulates extracellular protease secretion, was higher in LB-GP cultures than that in LB-G cultures. Additionally, sodium pyruvate increased acetate production in *S. aureus*, which helps maintain cell viability under acidic environment. In conclusion, pyruvate plays an important role in the survival and cytotoxicity of *S. aureus* under high glucose conditions. This finding may aid in the development of effective treatments for diabetic foot infections.

## 1. Introduction

*Staphylococcus aureus* is a commensal bacteria and opportunistic pathogen that causes a wide range of infections, from minor skin infections to severe life-threatening illnesses such as endocarditis and pneumonia (Tong et al., 2015). The severity of infections caused by *S. aureus* is influenced by various factors, including the host’s immune system, bacterial virulence, and environmental conditions (Scholthof, 2007). To adapt and survive in diverse infectious environments, *S. aureus* has evolved a complex regulatory system (Jenul and Horswill, 2019). For instance, the agr quorum-sensing system plays a crucial role in sensing changes in bacterial density and coordinating the production of virulence factors (Kong et al., 2006; Butrico and Cassat, 2020). Additionally, the cytoplasmic regulator of the sarA protein family is involved in regulating the production of virulence factors in response to environmental cues (Cheung et al., 2008).

Diabetes is a complex metabolic disorder that affects not only serum glucose level but also the levels of other sugars, such as fructose, mannose, and glucose-6-phosphate (G6P) (Menni et al., 2013). Excessive glucose intake has been shown to hinder the production of virulence factors and decrease the severity of *S. aureus* infections (Seidl et al., 2008; Dufresne et al., 2022). However, the high levels of sugars provide a unique metabolic environment that serves as a breeding ground for pathogens, leading to the development of diabetic foot infections (Seo et al., 2021). *S. aureus* is one of the most prevalent pathogens isolated from diabetic foot ulcers (DFUs) (Hawkins et al., 2022), indicating that the bacteria has adapted to the unique metabolic conditions of diabetes. Therefore, it is essential to comprehend the mechanism by which *S. aureus* adapts to the unique metabolic conditions of diabetes, which may help to develop effective treatments for diabetic foot infections.

Sugars are essential nutrients for the survival and growth of *S. aureus* within the host. Glucose is the main source of carbon and energy for the growth of *S. aureus*, but metabolism of other sugars, such as G6P, is also crucial. In fact, using these alternative sugars can actually increase the production of virulence factors, leading to more severe infections than with glucose metabolism alone (Seo et al., 2021). Moreover, pyruvate, the central metabolite of glycolysis, has been shown to increase the production of virulence factors and boost the pathogenicity of *S. aureus* (Harper et al., 2018). Recent research also suggests that pyruvate suppresses the *S. aureus* growth inhibition by betamethasone valerate, an anti-inflammatory drug used in the treatment of atopic dermatitis (Matsumoto et al., 2020). Exogenous pyruvate has been found to improve hyperglycemia, retinopathy and nephropathy (Chang et al., 2003; Hegde and Varma, 2005; Ju et al., 2012). Conversely, knockout of pyruvate kinase, an enzyme that catalyzes the conversion of phosphoenolpyruvate to pyruvate, has been shown to worsen diabetic nephropathy (Qi et al., 2017). Taken together, these findings suggest that pyruvate is important for ameliorating diabetes and diabetic-related complications. Despite the knowledge about pyruvate, the effect of this metabolite on the survival and cytotoxicity of *S. aureus* under high glucose conditions remains unclear. In this study, we found that the survival and cytotoxicity of *S. aureus* were greatly reduced under high glucose conditions, but which could be restored by the addition of sodium pyruvate.

## 2. Materials and methods

### 2.1. Bacterial strains and culture conditions

*Staphylococcus aureus* strain ATCC BAA-1717 (USA300) were kindly provided by Abace Biotechnology (Beijing, China), and which had remarkable hemolytic activity and pigment formation. The bacteria were routinely cultured at 37°C on 5% sheep blood agar plates (BA, Bio-caring, China) and then grown in lysogeny broth (LB, Solarbio Life Sciences, Beijing, China) at 37°C with shaking at 180 rpm. Overnight cultures of *S. aureus* strain BAA-1717 were diluted 1:100 into 3 mL fresh LB medium with 20 mM glucose (Sigma-Aldrich) or/and 20 mM sodium pyruvate (Sigma-Aldrich) in a 12 mL tube. All cultures were incubated at 37°C with shaking at 180 rpm, and the culture supernatants were collected at 24 h or 48 h post-inoculation. The cell-free culture medium (CFCM) was obtained by filtered through a PES filter (0.22 μm pore size; Millipore), used immediately or stored at −70°C.

### 2.2. Growth assays

Overnight cultures of *S. aureus* strain BAA-1717 were diluted 1:100 into a chemical defined medium (CDM, D6540, Solarbio Life Sciences, Beijing, China) or fresh LB medium with or without 5-, 10-, 20- or 40 mM sodium pyruvate. All cultures were incubated at 37°C with shaking at 180 rpm, either in or out of an anaerobic bag. After cultured for 24 h, the OD values were measured at 450 nm and the colony-forming units (CFUs) were determined by plating 5 μL serial dilutions on BA, then incubating them overnight at 37°C and counting the number of colonies. For the survival of *S. aureus* under acidic environment, overnight cultures of *S. aureus* strain BAA-1717 was added with 15 mM lactate (Sigma-Aldrich) or 15 mM acetate (Sigma-Aldrich), incubated at 37°C with shaking at 180 rpm for 48 h, and the CFUs was determined.

### 2.3. Quantitative hemolysis assays

Quantitative hemolysis assays were performed according to the methods described by Ridder et al. (2021). Briefly, discarded whole blood from healthy human subjects was washed twice with normal saline and then resuspended to a final concentration of 4% (v/v). Equal volumes of 4% human erythrocyte suspension and CFCM of *S. aureus* were added to 96-well flat-bottom plates and placed in 37°C incubator (static). The plate was centrifuged after incubated for 90 min. The supernatants were transferred to a new 96-well plate and measured at OD_450_ using a microplate reader. To inhibit extracellular protease, *S. aureus* strain BAA-1717 was grown in LB medium with 10 mM glucose or/and phenylmethylsulfonyl fluoride (PMSF, Sigma-Aldrich).

### 2.4. Measurement of neutrophils lysis

Peripheral blood was resuspended in RPMI 1640, layered with Ficoll Hypaque Plus (Sigma-Aldrich), and then centrifuged at 1000 *g* for 12 min. The red blood cell (RBC) pellet was incubated with red blood cell lysis buffer (CWBiotech, China) at a 9-fold volume for 15 min at 37°C to remove erythrocytes. After centrifugation at 1000 *g* for 10 min, the cell pellet was washed and resuspended in RPMI 1640 to the desired concentration. Neutrophil lysis was measured by lactate dehydrogenase (LDH) release assay. Briefly, the CFCM of *S. aureus* was added to 4.0 × 10^6^ neutrophils/mL to a total volume of 400 μL in 24-well plates and incubated at 37°C with 5% CO_2_. At the desired times, the samples were centrifuged at 3000 rpm for 5 min, and the supernatants were collected. The LDH activity in the culture supernatants was measured by using an automatic biochemical analyzer 7600 (Hitachi, Japan) according to the manufacturer’s instructions.

### 2.5. Measurement of pigmentation by methanol extraction

Measurement of pigmentation by methanol extraction was conducted as described by Sullivan and Rice (2021). Briefly, overnight cultures of *S. aureus* strain BAA-1717 was inoculated into 3 mL of LB medium with 20 mM glucose, 20 mM sodium pyruvate or both 20 mM glucose and 20 mM sodium pyruvate. After cultured for 24 h or 48 h, 1 mL of the cultures were centrifuged at 12000 rpm for 10 min. The resulting pellets were washed twice with normal saline, resuspended in 200 μL of methanol, and incubated for 30 min in an incubator with shaking at 180 rpm. The samples were then centrifuged again at 12000 rpm for 10 min, and the OD value of the supernatants was measured at 450 nm by using a microplate reader.

### 2.6. Gram-staining assays

Overnight cultures of *S. aureus* strain BAA-1717 were diluted 1:100 into fresh LB medium with 20 mM glucose, 20 mM sodium pyruvate or both 20 mM glucose and 20 mM sodium pyruvate. After cultured for 48 h, a smear of bacterial culture was air dried, heat fixed, and then stained with a commercial Gram-staining solution (BASO Diagnostics, China). Briefly, the staining process included 10 s in crystal violet solution, 10 s in iodine solution, a 20 s wash in decolorizer, then a final counter stain with safranin solution for 10 s. The stained slides were examined under a microscope (1000×).

### 2.7. Transmission electron microscopy assays

*S. aureus* strain BAA-1717 cultures were prepared by diluting overnight cultures 1:100 into fresh LB medium with 20 mM glucose, or both 20 mM glucose and 20 mM sodium pyruvate, and further cultured for 48 h. These cultures were collected and centrifugated at 12000 rpm for 10 min, washed twice with normal saline and then re-suspended in 2.5% glutaraldehyde solution with Millonig’s phosphate buffer. These samples were sent to the Transmission Electron Microscopy (TEM) Laboratory at the Department of Pathology, Xiangya Hospital for examination and photography using a Hitachi HT7700 electron microscope.

### 2.8. Lysostaphin lysis assays

Lysostaphin lysis assays were performed as previously reported with minor modifications (Grundling et al., 2006). Briefly, overnight cultures of *S. aureus* BAA-1717 were diluted 1:100 into fresh LB medium with 20 mM glucose, 20 mM sodium pyruvate or both 20 mM glucose and 20 mM sodium pyruvate, and cultured at 37°C, 180 rpm for 24 h. These cultures were collected and washed twice with PBS and resuspended to an OD_570_ of 1.8–2.1. This value was set as 100% at 0 min. Lysostaphin (Sigma-Aldrich) was added at final concentrations of 10 μg/mL, and the OD_570_ values were recorded at timed intervals, and data were plotted as percent OD_570_ values of the initial reading.

### 2.9. Aggregation assays

*S. aureus* strain BAA-1717 was grown in LB medium at 37°C with shaking at 180 rpm for overnight. For glucose-induced aggregation, overnight cultures were added with 20 mM glucose, 20 mM sodium pyruvate or both 20 mM glucose and 20 mM sodium pyruvate, and further cultured at 37°C for 7 h under static conditions. For lactate-induced aggregation, overnight cultures were pre-incubated with 30 mM or 60 mM sodium pyruvate for 1 h, then 15 mM lactate was added, and further cultured at 37°C for 7 h under static conditions. 150 μL of the supernatants was transferred to the wells of 96-well tissue culture plates, and the absorption was measured at 450 nm using a microplate reader. After cultured for 7 h, the medium was centrifuged at 12000 rpm for 10 min, and the content of glucose, lactate, pyruvate and total protein in the supernatants were analyzed by an automatic biochemical analyzer 7600 according to the manufacturer’s instructions. The content of acetate in the supernatants was analyzed by Acetate Colorimetric Assay Kit (Sigma-Aldrich) according to the manufacturer’s instructions.

### 2.10. RNA isolation and real-time RT-PCR

*S. aureus* strain BAA-1717 were grown in LB medium supplemented with 20 mM glucose, 20 mM sodium pyruvate or both 20 mM glucose and 20 mM sodium pyruvate, and incubated at 37°C with shaking at 180 rpm. After cultured for 6 h or 24 h, bacterial cells were collected and centrifuged at 12000 rpm for 10 min. The cell pellets were re-suspended in 200 μL PBS with 10 μg/mL lysostaphin and incubated at room temperature for 30 min. Total RNA was extracted and purified by using nucleic acid extraction kit (paramagnetic particle method) (Shanghai BioGerm Medical Technology Co., Ltd.) according to the manufacturer’s instructions. The RNA quality and concentration were evaluated by using a NanoDrop 1000 (Thermo Fisher Scientific). Then, DNA was removed by DNase I, and then total RNA was reverse transcribed to cDNA using a reverse transcription kit (TransGen Biotech, Beijing, China) according to the manufacturer’s instructions. The gyrB gene was used as an internal reference to normalize the expressions of genes of interest and PCRs were performed in 50 μL reaction mixtures. The relative quantification method (2^-△△Ct^) was used to analyze the transcription level of target genes. The primers used in this study were reported in previous study (Abdelhady et al., 2014; Shi et al., 2019; Shang et al., 2022). All analyses were conducted in triplicate.

### 2.11. Statistical analysis

Statistical analysis was performed with GraphPad Prism software version 8.3. Significance levels were calculated by using one way ANOVA or two-way ANOVA analysis. Statistical significance was defined as *p* < 0.05. All error bars depict the standard deviation. Each experiment was repeated at least three times.

## 3. Results

### 3.1. Sodium pyruvate increases the cytotoxicity of *S. aureus* to human erythrocytes and neutrophils

**Figure 1:**
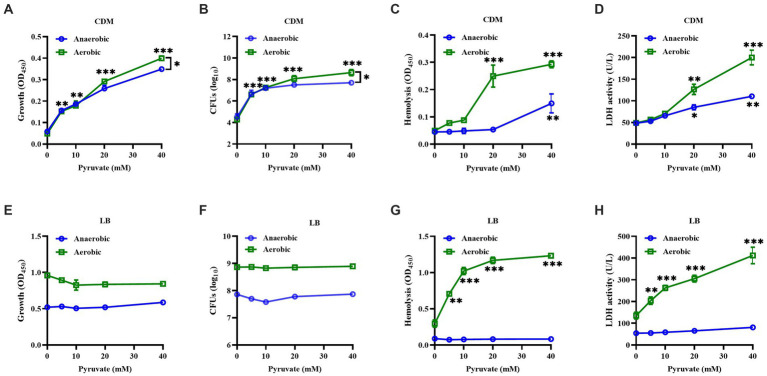
Effect of sodium pyruvate on the growth and hemolytic activity of *S. aureus*. *S. aureus* grown in CDM medium with different concentration of sodium pyruvate. After cultured for 24 h, the turbidity was measured **(A)**, the CFUs were determined by serial dilutions **(B)**, and the cytotoxicity of culture supernatants to human erythrocytes and neutrophils was analyzed **(C,D)**. *S. aureus* was grown in LB medium with different concentration of sodium pyruvate, and the turbidity was measured **(E)**, the CFUs were determined by serial dilutions **(F)**, and the cytotoxicity of culture supernatants to human erythrocytes and neutrophils was analyzed **(G,H)** after being cultured for 24 h. ^*^ *p* < 0.05, ^**^ *p* < 0.01, ^***^ *p* < 0.001.

*S. aureus* strain BAA-1717 was firstly grown in a CDM medium without carbon sources under either aerobic or anaerobic conditions, and sodium pyruvate was added as the only carbon source available to the bacteria. The growth of *S. aureus* was significantly increased by sodium pyruvate under both anaerobic and aerobic conditions, with a higher tendency observed under aerobic condition (Figures 1A,B). Subsequently, the cytotoxicity of *S. aureus* was analyzed *in vitro*, and found that the cytotoxicity of *S. aureus* CFCM to human erythrocytes and neutrophils was significantly increased by sodium pyruvate under both anaerobic and aerobic conditions (Figures 1C,D). To further investigate the effect of pyruvate on the cytotoxicity of *S. aureus*, then the bacteria was grown in LB medium supplemented with different concentrations of sodium pyruvate. Unlike the ascending trend observed in CDM, the growth of *S. aureus* in LB medium was unaffected by sodium pyruvate under both anaerobic and aerobic conditions (Figures 1E,F). However, the cytotoxicity of *S. aureus* CFCM to human erythrocytes and neutrophils was significantly increased by sodium pyruvate under aerobic conditions, which remained extremely low under anaerobic conditions (Figures 1G,H). These data indicate that sodium pyruvate increases the cytotoxicity of *S. aureus* under aerobic conditions *in vitro*.

### 3.2. Sodium pyruvate restores the reduced cytotoxicity of *S. aureus* under high glucose conditions

**Figure 2:**
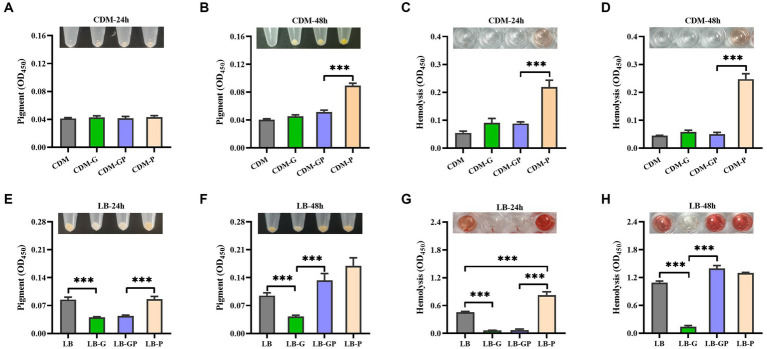
Effect of sodium pyruvate on the cytotoxicity of *S. aureus* under high glucose conditions. *S. aureus* strain BAA-1717 was grown in CDM, CDM-G, CDM-GP, and CDM-P medium. After cultured for 24 h or 48 h, the pigment formation was determined **(A,B)**, and the hemolytic activity of CFCM was analyzed **(C,D)**. *S. aureus* strain BAA-1717 was grown in LB, LB-G, LB-GP and LB-P medium. After cultured for 24 h or 48 h, the pigment formation was determined **(E,F)**, and the hemolytic activity of CFCM was analyzed **(G,H)**. ^***^ *p* < 0.001.

*S. aureus* is the most described Gram-positive pathogen in DFUs (Macdonald et al., 2021), whether pyruvate affects the cytotoxicity of *S. aureus* under high glucose conditions is unclear. *S. aureus* strain BAA-1717 was firstly grown in CDM medium with high glucose and sodium pyruvate, and found that the pigment formation was significantly increased in the CDM-P culture after cultured for 48 h, but no significant difference was observed between CDM-G and CDM-GP cultures (Figures 2A,B). The hemolytic activity of *S. aureus* remained extremely low in CDM, CDM-G and CDM-GP cultures, but was higher in CDM-P cultures (Figures 2C,D). Next, *S. aureus* was grown in LB medium with glucose and sodium pyruvate, and found that both pigment formation and hemolytic activity were significantly decreased by high glucose, which was not affected by the addition of sodium pyruvate after being cultured for 24 h (Figures 2E,G). As the culture time increased to 48 h, the pigment formation and hemolytic activity of *S. aureus* grown in LB-GP medium was greatly increased and restored to the levels of LB and LB-P cultures (Figures 2F,H). These data indicate that the reduced hemolytic activity and pigment formation of *S. aureus* under high glucose conditions is restored by sodium pyruvate.

### 3.3. Sodium pyruvate restores the reduced cytotoxicity of *S. aureus* under high glucose conditions by up-regulation of sarA expression

**Figure 3:**
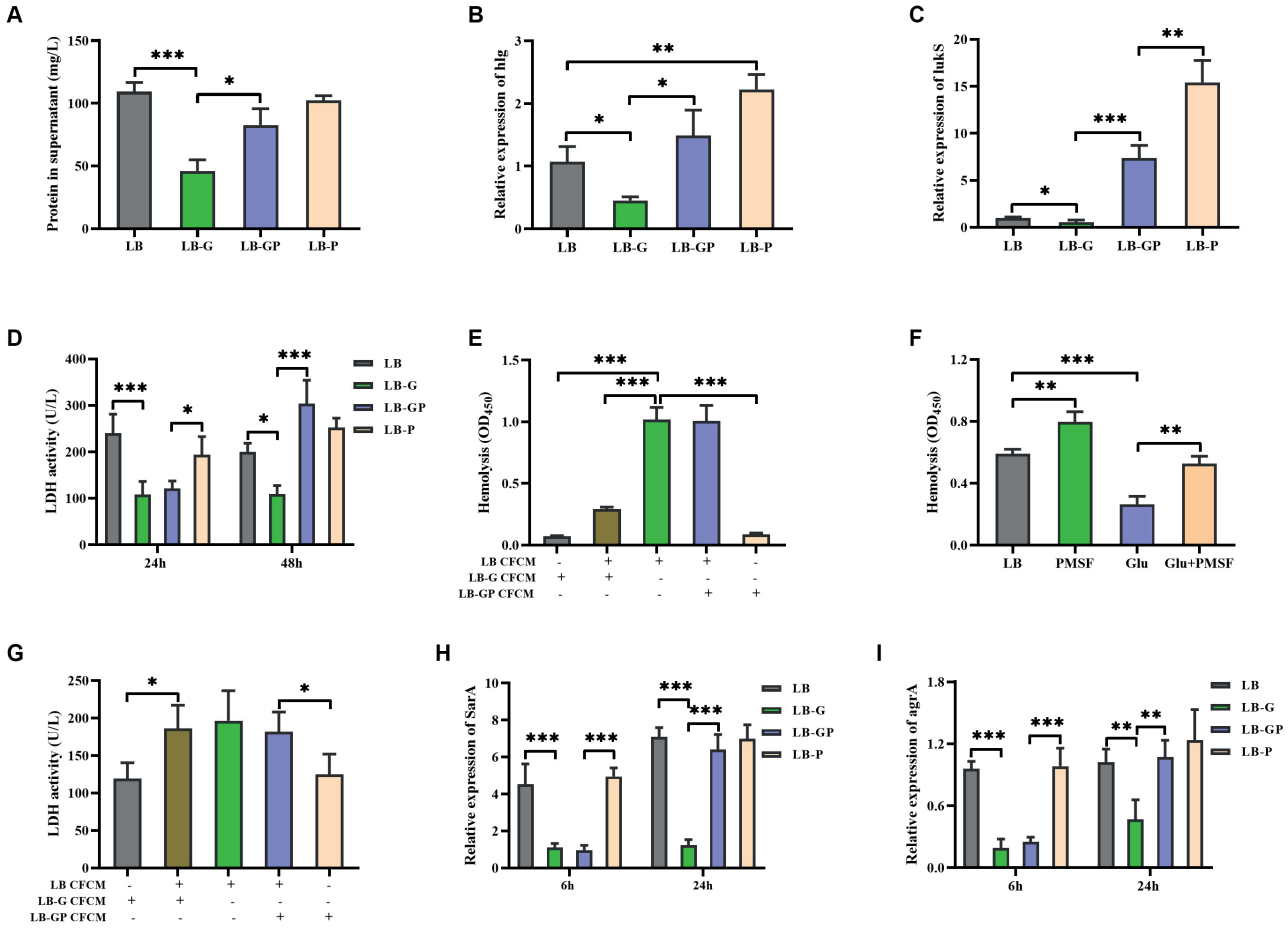
Sodium pyruvate affects the production of extracellular protease under high glucose conditions. Overnight cultures of *S. aureus* strain BAA-1717 was diluted 1:100 into LB, LB-G, LB-GP, and LB-P medium. **(A)** the total protein in the culture supernatants was detected after being cultured for 24 h. The expression of hlg **(B)** and lukS **(C)** was examined after being cultured for 6 h. **(D)** The cytotoxicity of culture supernatants to human neutrophils was examined. **(E)** The CFCM of LB cultures were pre-incubated with the CFCM of LB-G or LB-GP cultures, and the cytotoxicity to human erythrocytes was examined. **(F)** Lysis of human erythrocytes by the CFCM of *S. aureus* when grown in medium with glucose or/and PMSF. **(G)** The CFCM of LB cultures were pre-incubated with the CFCM of LB-G or LB-GP cultures, and the cytotoxicity to human neutrophils was examined. The expression of sarA **(H)** and agrA **(I)** was examined after being cultured for 6 h or 24 h. ^*^ *p* < 0.05, ^**^ *p* < 0.01, ^***^ *p* < 0.001.

Pyruvate has been shown to induce the production of extracellular proteins and virulence factors, such as Panton-Valentine Leucocidin (PVL), resulting in increased virulence of *S. aureus* (Harper et al., 2018). As shown in Figure 3A, the total protein in LB-GP cultures was higher than that in LB-G cultures, which was comparable to the level of LB and LB-P cultures. The expression of hlg and lukS (a PVL encoding gene) in LB-GP cultures was higher than that in LB-G cultures (Figures 3B,C). PVL has been shown to disrupt the body’s defense system by lysing human polymorphonuclear cells (Loffler et al., 2010). However, the reduced cytotoxicity of *S. aureus* CFCM to human erythrocytes and neutrophils by high glucose was not affected by the addition of sodium pyruvate after being cultured for 24 h (Figures 2G, 3D). As the culture time increased to 48 h, the cytotoxicity of *S. aureus* CFCM from LB-GP cultures to human neutrophils was significantly increased compared to LB-G cultures (Figure 3D). Additionally, the cytotoxicity of LB culture CFCM to human erythrocytes was significantly decreased when pre-incubated with the CFCM of LB-G cultures, but unaffected by the CFCM of LB-GP cultures (Figure 3E). Additionally, the reduced hemolytic activity of *S. aureus* by high glucose could be restored by PMSF, a serine protease inhibitor (Figure 3F). However, the cytotoxicity of LB culture CFCM to human neutrophils was unaffected when pre-incubated with the CFCM of LB-G or LB-GP cultures (Figure 3G). These data indicate that high levels of extracellular proteases are present in the CFCM of LB-G cultures, which degrade the hemolytic factors of *S. aureus*. Previous studies have shown that the production of extracellular proteases is negatively regulated by sarA (Karlsson and Arvidson, 2002; Ramirez et al., 2020). Next, the expression of sarA was examined by real-time RT-PCR, and found that the expression of sarA was lower in both LB-G and LB-GP cultures than that of LB and LB-P cultures after being cultured for 6 h. As the culture time increased to 24 h, the expression of sarA in LB-GP cultures was greatly increased and restored to the levels of LB and LB-P cultures (Figure 3H). The expression of agrA was lower in LB-G cultures, which was greatly increased by addition of sodium pyruvate (Figure 3I). Therefore, sodium pyruvate reduces the production of extracellular protease, leading to restore the reduced cytotoxicity of *S. aureus* under high glucose conditions.

### 3.4. Sodium pyruvate restored the reduced survival of *S. aureus* under high glucose conditions

**Figure 4:**
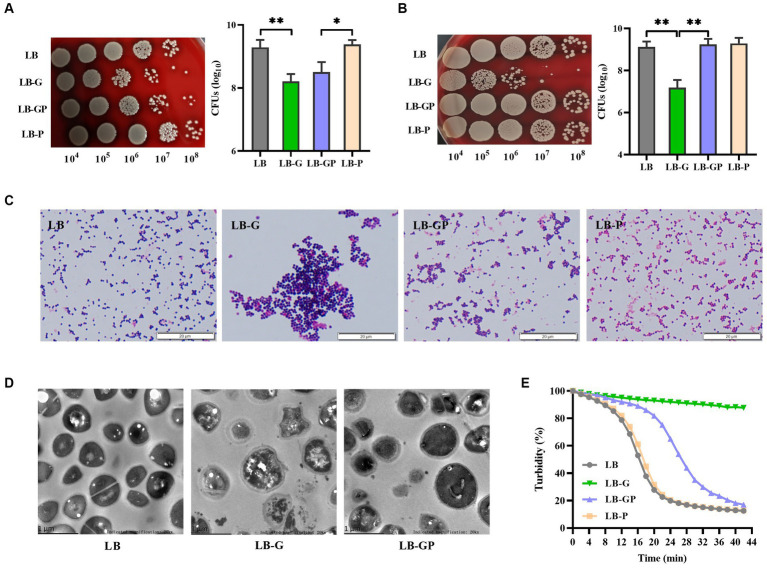
Effect of sodium pyruvate on the survival of *S. aureus* under high glucose conditions. *S. aureus* was grown in LB, LB-G, LB-GP and LB-P medium, and the CFUs were determined after being cultured for 24 h **(A)** or 48 h **(B)**. After cultured for 48 h, bacterial cells were analyzed by Gram-staining **(C)** and TEM assays **(D)**. **(E)** After cultured for 24 h, bacterial cells were collected and lysed by 10 μg/mL lysostaphin, and the value of OD_570_ was taken at timed intervals. ^*^ *p* < 0.05, ^**^ *p* < 0.01.

As shown in Figure 4A, the CFUs of *S. aureus* grown in LB-G and LB-GP medium were reduced nearly 10-fold compared to LB and LB-P cultures, and no significant difference was observed between LB-G and LB-GP cultures after being cultured for 24 h. As the culture time increased to 48 h, the CFUs of *S. aureus* from LB-G cultures decreased by more than 100-fold. In contrast, the CFUs of *S. aureus* from LB-GP cultures were greatly increased and returned to the levels of LB and LB-P cultures (Figure 4B). Next, Gram-staining assay was used to examine the morphologic changes of *S. aureus*. The bacteria from LB-G cultures were more aggregated and larger in size than that of LB cultures, which was significantly alleviated in the LB-GP cultures (Figure 4C). TEM assays were used to further determine the structural changes of *S. aureus* affected by sodium pyruvate under high glucose conditions, and found that the cells from LB-G cultures were slightly larger in size and had more compact cytoplasm than those from LB and LB-GP cultures (Figure 4D). Previous studies have shown that *S. aureus* grown in rich-medium (high glucose) is highly resistant to lysostaphin (Wu et al., 2019; Luo et al., 2020). Herein, *S. aureus* from LB-G cultures was highly resistance to lysostaphin, which was greatly attenuated when grown in LB-GP medium (Figure 4E). These data indicate that sodium pyruvate restores the survival of *S. aureus* under high glucose conditions by supporting a second round of growth during stationary phase.

### 3.5. Sodium pyruvate enhances the production of acetate in *S. aureus* under high glucose conditions

**Figure 5:**
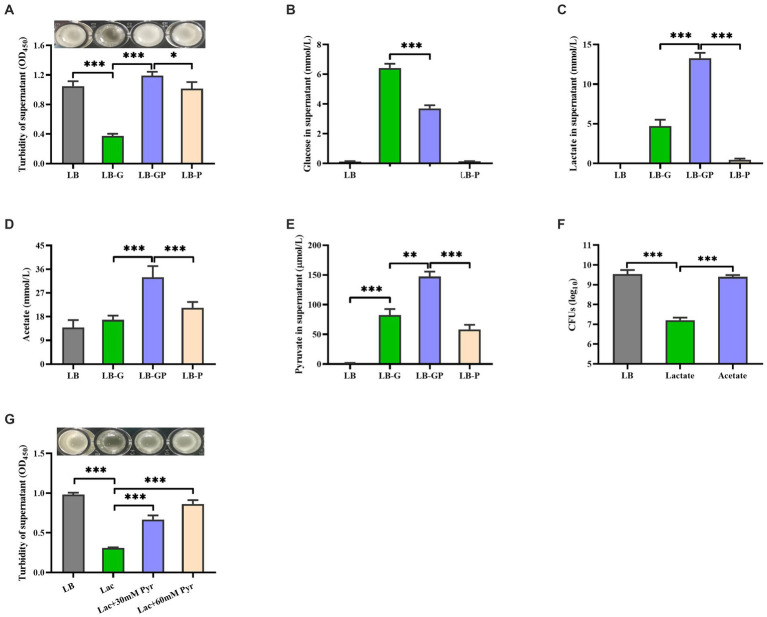
Sodium pyruvate altered glucose metabolism of *S. aureus* under high glucose conditions. Overnight cultures of *S. aureus* were added with 20 mM glucose, 20 mM sodium pyruvate or both 20 mM glucose and 20 mM sodium pyruvate. After incubated for 7 h, the turbidity was determined **(A)**, and the levels of glucose **(B)**, lactate **(C)**, acetate**(D)**, and pyruvate **(E)** in the culture supernatants were detected. **(F)** Overnight cultures of *S. aureus* were incubated with lactate or acetate, and the CFUs was determined after incubation for 48 h. **(G)** Overnight cultures of *S. aureus* were pre-incubated with 30 mM or 60 mM sodium pyruvate for 1 h, and then the aggregation was induced by 15 mM lactate. ^*^ *p* < 0.05, ^**^ *p* < 0.01, ^***^ *p* < 0.001.

Our previous study has shown that high glucose induces aggregation of *S. aureus* (Luo et al., 2019). We investigate whether pyruvate affects the aggregation of *S. aureus* under high glucose conditions. As shown in Figure 5A, sodium pyruvate alone did not affect *S. aureus* aggregation, but the aggregation of *S. aureus* induced by high glucose was greatly inhibited by the addition of sodium pyruvate. Subsequently, the glycolytic metabolites in the culture supernatants were analyzed. Glucose was undetected in the LB and LB-P cultures, which was higher in the LB-G cultures compared to LB-GP cultures (Figure 5B). The levels of lactate, acetate and pyruvate were higher in the LB-GP cultures compared to LB-G cultures (Figures 5C-E). The content of acetate in the supernatants of LB-GP cultures was as high as 30 mM, which is much higher than that of lactate (13 mM). Inactivation of the Pta-AckA pathway significantly reduces the production of acetate, resulting in reduced growth rate and viability of *S. aureus* (Sadykov et al., 2013; Marshall et al., 2016). The CFUs of stationary-phase *S. aureus* strain BAA-1717 was significantly decreased by the addition of lactate, but was unaffected by acetate (Figure 5F). Additionally, high level of lactate induced the aggregation of *S. aureus*, which was also significantly inhibited by pre-incubation with sodium pyruvate (Figure 5G). These data indicate that sodium pyruvate enhances the production of acetate, maintaining the survival of *S. aureus* under high glucose conditions.

## 4. Discussion

High glucose-induced cytotoxicity to eukaryocytes and pathogens has been widely recognized (Gao et al., 2015; Cao et al., 2019; Luo et al., 2020; Zhang S. et al., 2020). Pyruvate has been shown to protect the development and progression of diabetic nephropathy (Ju et al., 2012). Meanwhile, pyruvate alleviates endothelial cell injury and maintains cell viability under high glucose conditions (Zhang X. M. et al., 2020; Yako et al., 2021). In this study, we investigated the effect of pyruvate on *S. aureus* under high glucose conditions, and found that sodium pyruvate was able to reverse the reduced survival and cytotoxicity of *S. aureus* under high glucose conditions by enhanced acetate production.

The virulence of *S. aureus* largely depends on its metabolic pathways, including glycolysis, pentose phosphate pathway, and tricarboxylic acid (TCA) cycle pathway (Lan et al., 2010; Richardson, 2019). Carbohydrates are mainly broken down by the glycolytic and pentose phosphate pathways, but the TCA cycle activity of *S. aureus* is mostly inhibited when nutrients are abundant (Strasters and Winkler, 1963). Pyruvate, a key metabolite of glycolysis, can be further broken down through the TCA cycle to produce energy for growth. In this study, sodium pyruvate served as the solely carbon source promoted the growth of *S. aureus* in a CDM medium, but which was not observed when grown in LB medium (Figure 1). In addition to produce energy, pyruvate also plays a critical role in the production of virulence factors that allow *S. aureus* to cause infection (Harper et al., 2018). We observed that the pigment formation and cytotoxicity of *S. aureus* was greatly increased by sodium pyruvate when grown in both CDM and LB medium under aerobic condition. These data suggest that pyruvate is an important carbon source for the cytotoxicity of *S. aureus*, which is consistent with a previous study (Harper et al., 2018).

In patients with diabetes, high level of serum glucose can lead to an increase in pyruvate production (Anderson and Marks, 1962), and high level of pyruvate was detected in the *S. aureus* culture with high glucose (Figure 5E). Additionally, pyruvate has been shown to enhance the growth and virulence of certain bacterial pathogens, including *S. aureus and Pseudomonas aeruginosa* (Petrova et al., 2012; Harper et al., 2018). Our unpublished data found that *S. aureus* isolated from DFUs have higher cytotoxicity to human erythrocytes than those isolated from non-diabetic sepsis and wounds. In this study, high glucose greatly reduced the pigment formation and cytotoxicity of *S. aureus* when grown in LB medium, which was restored by the addition of sodium pyruvate, but this phenomenon was not observed in the CDM medium. High glucose has been shown to reduce the production of virulence factors by *S. aureus in vitro* (Regassa et al., 1992; Seidl et al., 2008; Dufresne et al., 2022), but hyperglycemia makes individuals more susceptible to developing severe *S. aureus* infection (Thurlow et al., 2020; Butrico et al., 2023). These data indicate that pyruvate may be involved in the pathogenic process of *S. aureus* induced diabetic foot infections.

The fate of pyruvate is largely determined by the redox state of the cell (Troitzsch et al., 2021). In this study, sodium pyruvate was quickly utilized by *S. aureus* when grown in the LB medium and only a small amount of lactate was detected. *S. aureus* has been shown to mainly secret acetate in the presence of pyruvate, which increases the activity of Pta-AckA pathway (Troitzsch et al., 2021). These data suggest that sodium pyruvate may be converted into acetate or acetyl-CoA, and excreted to the culture medium. The content of acetate in the supernatants of LB-GP cultures was higher than that of LB-G and LB-P cultures (Figure 5D). During aerobic growth on carbohydrates, only a small amount of pyruvate actually enters the TCA cycle, because the genes encoded TCA-cycle enzymes are repressed by CcpA in presence of glucose (Seidl et al., 2009). Consequently, the reduced survival and cytotoxicity of *S. aureus* under high glucose conditions was not affected by sodium pyruvate at the exponential phase due to the presence of glucose. Once glucose in the medium is exhausted, the repression of the TCA cycle by CcpA is lifted, and these excreted metabolites can be reassimilated (Somerville et al., 2003). We observed that the rate of glucose consumption by *S. aureus* was faster in LB-GP cultures than that of LB-G cultures, suggesting that *S. aureus* grown in LB-GP medium was more likely to switch from pyruvate secretion to pyruvate import from the surrounding environment. Additionally, *S. aureus* is able to utilize excreted metabolites as carbon sources, which supports secondary round of growth during stationary phase (Patton et al., 2005). We observed that the survival of stationary-phase *S. aureus* was significantly reduced in the acidic environment induced by lactate, while it was unaffected by acetate (Figure 5F). Therefore, sodium pyruvate restores the reduced survival and cytotoxicity of *S. aureus* under high glucose by supporting secondary round of growth during stationary phase.

The aggregation *S. aureus* was more apparent in LB-G cultures than that in LB-GP cultures. According to a recent study reported by Kinney et al., the formation of vegetation in *S. aureus* infective endocarditis is inversely correlates with sarA expression (Kinney et al., 2022). SarA is also involved in the regulation of secreted enzymes and toxins, and mutation of sarA in the USA300 strain LAC increases overall protease activity and decreases the abundance of α-toxin (Chan and Foster, 1998; Karlsson and Arvidson, 2002; Ramirez et al., 2020). We observed that the total protein in LB-G cultures was lower than that in LB-GP cultures, and the hemolytic activity of *S. aureus* was greatly reduced when pre-incubated with the CFCM of LB-G cultures. In addition, the reduced hemolytic activity of *S. aureus* was restored by PMSF, a serine protease inhibitor. These data indicate that the hemolytic factors are degraded by the CFCM of LB-G cultures. The expression of sarA and agrA was significantly decreased by high glucose, which was restored by sodium pyruvate at stationary phase. Pyruvate has been shown to induce the production of virulence factors by activating the *S. aureus* master regulators Agr and SaeRS (Harper et al., 2018). Therefore, sarA and AgrA are involved in restoring the reduced cytotoxicity of *S. aureus* under high glucose conditions by the addition of sodium pyruvate.

Intermediate metabolites of glycolysis, such as pyruvate and G6P, which is highly present in diabetes, have been shown to induce expression of staphylococcal virulence factors that cause severe tissue necrosis and bacterial burden in skin infections (Harper et al., 2018; Seo et al., 2021). Therefore, it is possible to disrupt the pathogenesis of *S. aureus* under high glucose conditions by targeting pyruvate metabolism, thereby improving patient outcomes.

## Data availability statement

The raw data supporting the conclusions of this article will be made available by the authors, without undue reservation.

## Ethics statement

This study was approved by the Ethics Committee of the Third Xiangya Hospital of Central South University. For peripheral blood used in this study, informed consent was obtained from all healthy donors.

## Author contributions

TC, HX, and ZL designed the study, analyzed the results, and wrote and reviewed the manuscript. TC, HX, XY, and ZL conducted the experiments. All authors contributed to the article and approved the submitted version.

## Funding

This study was supported by Natural Science Foundation of Hunan Province (grant no. 2023JJ30837). The funding body had no role in the design of the study and collection, analysis, and interpretation of data and writing the manuscript.

## Conflict of interest

The authors declare that the research was conducted in the absence of any commercial or financial relationships that could be construed as a potential conflict of interest.

## Publisher’s note

All claims expressed in this article are solely those of the authors and do not necessarily represent those of their affiliated organizations, or those of the publisher, the editors and the reviewers. Any product that may be evaluated in this article, or claim that may be made by its manufacturer, is not guaranteed or endorsed by the publisher.
